# Cytomegalovirus in donors for fecal microbiota transplantation, the phantom menace?

**DOI:** 10.1371/journal.pone.0287847

**Published:** 2023-06-29

**Authors:** Tatiana Galpérine, Ilka Engelmann, Sebastien Hantz, Déborah Postil, Anny Dewilde, Dominique Deplanque, Renaud Martin, Julien Labreuche, Mouna Lazrek, Stéphanie Somers, Elodie Ribot, Sophie Alain

**Affiliations:** 1 Service of Infectious Diseases, Lausanne University Hospital, Lausanne, Switzerland; 2 French Group of Fecal Microbiota Transplantation (GFTF), France; 3 Service of Infectious Diseases, CHU Lille France, Lille, France; 4 Laboratoire de Virologie URL3610, Lille, France; 5 Laboratoire de Bactériologie-Virologie-Hygiène, National Reference Center for Herpesviruses (NRCHV), CHU Limoges, Limoges, France; 6 Direction of Research and Innovation, CHU de Limoges, Limoges, France; 7 CIC 1403-Centre d’Investigation Clinique, Univ. Lille, Inserm, CHU Lille, Lille University, Lille, France; 8 Santé Publique: Épidémiologie et Qualité des Soins, CHU Lille, University of Lille, Lille France; Aga Khan University, PAKISTAN

## Abstract

**Background:**

Fecal Microbiota Transplantation (FMT) has become the preferred treatment for recurrent *Clostridioides difficile* Infections (CDI). However, donor screening is a complex process that varies between countries. The primary objective of screening is to prevent the transfer of potential pathogens from the donor to the recipient via feces. Many guidelines recommend Cytomegalovirus (CMV) testing as part of donor screening, but is the risk of CMV transmission well supported by evidence?

**Materials/methods:**

A French prospective cross-sectional multicenter single-arm study estimated the frequency of detection of CMV in the stool of voluntary healthy donors selected for FMT. All preselected donors were tested for CMV antibodies in blood, and if positive, CMV DNA PCR was performed on whole blood and stool. For samples CMV positive in stool PCR, or case of serological markers positive for IgM, we planned isolation of CMV in cell culture.

**Results:**

From June 1, 2016, to July 31, 2017, 500 healthy donors (250 per center) were recruited and 483 included. Of these, 301 were CMV seronegative, and 182 tested positive for CMV IgM and/or IgG. Stool CMV PCR was performed in 162 donors. In two cases, the initial analysis was positive, but below the limit of quantification. Repeated PCR tests using Siemens and Altostar assays were negative. No infectious CMV could be detected in cell culture of these two samples and in the stool of 6 CMV IgM-positive donors.

**Conclusions:**

Our study shows that healthy volunteers with positive CMV serology do not shed CMV DNA in their stool, as detected by PCR or cell culture. This study provides another argument to remove CMV screening for FMT donors.

## Introduction

Fecal microbiota transplantation (FMT) involves transferring minimally manipulated stool from a healthy donor into a patient’s gastrointestinal tract to restore gut microbiome homeostasis. However, the selection of donors for FMT can be challenging. Several publications have shown a low rate of donor eligibility (2–10%) [1–5]. The transmission of an extended-spectrum β-lactamase-producing (ESBL) *Escherichia coli* strain from donor feces to two immunocompromised patients highlighted the critical role of donor screening [6]. However, if ESBL-producing pathogen detection is mandatory in a screening panel, several other recommended tests rely only on potential safety issues. Many consensus experts recommend performing serological testing for cytomegalovirus (CMV) (donor and recipient) as part of the screening panel [7, 8]. The donor must have negative serology for CMV if the recipient is CMV negative. This recommendation adds additional steps to an already complex process, eliminates potential donors, and increases costs even though the risk of transmission by FMT has never been proven to date.

The main objective of our study is to assess the presence of CMV and, if detected, the infectivity of CMV in stool samples from healthy volunteers with documented positive CMV serology who have been selected as potential donors for FMT.

## Materials and methods

### Study design

We conducted a prospective, cross-sectional, multicenter, single-arm study to measure the frequency of CMV detection in stool samples from healthy volunteers selected as universal fecal donors for FMT, with a positive CMV serology. The study was conducted at two clinical trial units (CTUs) located at Lille and Limoges university hospitals from June 1, 2016, to July 31, 2017.

### Study approval and registration

This study was approved by the French National Agency for Medicines and Health Products Safety and was registered on ClinicalTrials.gov (Number: NCT02694484). The study adheres to ethical principles as outlined in the declaration of Helsinki and follows all regulations of the International Conference of Harmonization Good. All participants provided written informed consent. Ethics approval was obtained from the regional ethics committee (2015-A01468-41).

### Participants

All volunteers were enrolled through a national CTUs register. A pre-screening selection was carried out by phone using a standardized questionnaire to check main clinical eligibility criteria (see S1 File). CMV serology status is usually unknown and cannot be a pre-selection criterion. We included healthy volunteers aged between 18 and 65 years old with regular intestinal transit (without chronic constipation, neither acute nor chronic diarrhea, nor irritable bowel syndrome) and an average body mass index (BMI) of less than 30 and greater than 16.9. Volunteers had to provide signed informed consent. At inclusion, each volunteer had a CMV serology test. Volunteers were excluded if they had been exposed to anti-CMV treatment within three months before inclusion or if absolute exclusion criteria defined by the French Group Fecal Transplant guidelines existed [9]. Volunteers were also required to avoid urine or blood contamination of the stool for laboratory analysis. In the case of CMV seropositivity, we obtained additional blood and stool samples on the next visit within 30 days after inclusion. The study involved two visits: inclusion and follow-up, as well as a stool sample and two blood tests.

### CMV serology

CMV serology was performed using the LIAISON^TM^ CMV IgG II and LIAISON^TM^ CMV IgM II assays on the LIAISON^TM^ XL analyzer (Diasorin, Saluggia, Italie). Results were reported as negative for CMV IgG specimens with <12 U/ml, indeterminate between12 to 14 U/ml, and positive if >14 U/ml. For CMV IgM detection, results were reported as negative for samples with <18 U/ml, indeterminate between18 to 22 U/ml, and positive if >22 U/ml.

### Nucleic acid extraction and detection of CMV DNA in whole blood specimens

Whole blood specimens were obtained in EDTA tubes within 30 days after inclusion. Nucleic acid extraction was done using Versant kPCR Molecular systems SP using the Versant sample preparation 1.2 Reagents (Siemens Healthcare Diagnostics, France) according to the manufacturer’s instructions. 400-μl of whole blood was mixed with 400-μl buffer. 475 μl of the specimen/buffer mixture were used for automatic extraction. Quantitative CMV DNA detection was done using the kPCR PLX^TM^ CMV DNA assay (Siemens Healthcare Diagnostics, France) according to the manufacturer’s instructions. Briefly, 5.5 μl Master A and 16.5 μl Master B were mixed with 11 μl of nucleic acids and cycling was performed on Versant kPCR Molecular systems AD (Siemens Healthcare Diagnostics, France). The assay includes an internal control to check for extraction quality and PCR inhibition.

### Nucleic acid extraction and detection of CMV DNA in stool specimens

The laboratory rejected a stool specimen contaminated with macroscopic blood or urine. Stool specimens were placed at +4°C and sent to the laboratory less than 6 hours after stool emission for preparation of 1g aliquots stored at -80°C until analysis. The method for stool pretreatment before total DNA extraction was first optimized using a calibrated virion stock used as external quality control for routine CMV Rgene assay at the reference center (batch of clarified supernatant from large-scale cell culture of the Towne HCMV reference strain, highly reproducible on Levey-Jennigs follow-up. The stock was diluted in CMV-negative volunteer stool to a final concentration of 10^6^ IU/mL of stool and submitted before extraction to either pretreatment with the BMX stool device (BioMérieux France) or to our routine method for DNA viruses in the stool (1g of stool was dissolved in 800μL of Nuclisens^TM^ (BioMérieux, France) buffer). It was then mixed thoroughly with a vortex, incubated for 10 min at room temperature, and centrifuged for 30 min at 3000g. Nucleic acid extraction was performed on 400μL of supernatant with the Nuclisens^TM^Easy Mag method (BioMérieux, France), protocol-specific B. CMV genome copies were quantified per ng of total DNA by real-time PCR with the commercial CEIVD CMV R-gene™ kit (BioMérieux, France). An internal control provided in the kit was added at extraction and co-amplified to check for both extraction quality and PCR inhibitors. If this internal control was not at the expected value, the result was excluded, and a new aliquot of the stool was tested. If the presence of an inhibitor was confirmed, the sample was excluded from the analysis.

According to dilutions of the WHO standard in stool, the limit of detection of the method was 500 IU of CMV genome per nanogram of total DNA.

All positive stool results were checked at Lille university hospital using two different methods, the Versant® CMV PCR (Siemens, healthcare) and the AltoStar® CMV PCR Kit 1.5 (Altona diagnostics) with the same extraction method. The final result was considered positive if all the RT-PCR were positive. In the case of discordant results between the two centres, we interpreted it as negative if two control PCR were negative.

### Isolation of CMV in cell-culture

Isolation of CMV from stools was performed from 2x1g of PCR-positive stools on MRC-5 human embryonic fibroblasts (BioMérieux, France). Stools were thawed at 37°C in 2 tubes containing 3ml of medium (MEM with Earle’ salts, Eurobio, Courtaboeuf, France) each. After vortexing thoroughly, the supernatant was clarified by centrifugation (3500g, 30min), then filtered on a 0.45uM membrane and added with 60uL of antibiotics (10000U/mL penicillin, 16mg/mL gentamicin, 500mg/mL colimycin). The rapid culture was performed on 24-well plates by inoculating 500 microliters of stool supernatant onto six wells and centrifugation at 37°C 3500g 45 minutes to enhance virus adhesion and penetration. The inoculum was then replaced by culture medium (ME-Earle’s plus Lglutamin and NaHCO3, with 10% fetal calf serum 0.1M Hepes (all from Eurobio, Coutaboeuf France) added with 50mg/mL gentamicin, 50U/mL penicillin). Plates were incubated for 48h at 37°C in the presence of 5% CO_2._ Infectious viruses were identified by immunohistochemistry using mouse anti-E13 antibodies at 1/40 in phosphate buffer saline (PBS) (BioMérieux Rgene, Varilhes, France) and peroxydase-coupled goat anti-mouse antibodies 1/100 in PBS (Gibco) as previously described. In parallel, 1ml of supernatant was inoculated onto MRC-5 cells seeded in a 25cm^2^ flask, the inoculum was removed after 3 hours, and incubation was prolonged for three weeks with a daily observation of cytotoxicity and cytopathic effect on an inverted microscope, medium renewal twice a week and cell-passage every week.

### Outcomes

The primary objective was to determine the prevalence of CMV DNA detection in stool samples of healthy volunteers who met the FMT donor selection criteria and had a positive CMV serology.

### Statistical analysis

Quantitative variables were expressed as mean (standard deviations) or medians (interquartile range), and categorial variables were expressed in numbers and percentages. The normality of the numerical parameters was checked graphically and tested using the Shapiro-wilk test. Fisher’s exact test and Chi-2 test were used as appropriate. For the correlations between log_10_ CMV DNA in serum and feces, we used the Spearman correlation coefficient. All tests were two-sided, and p values of 0.05 or less were considered significant. Data were analyzed using SAS software (version 9.3, SAS Institute Inc., Cary, NC, USA).

## Results

### Patients

**Fig 1:**
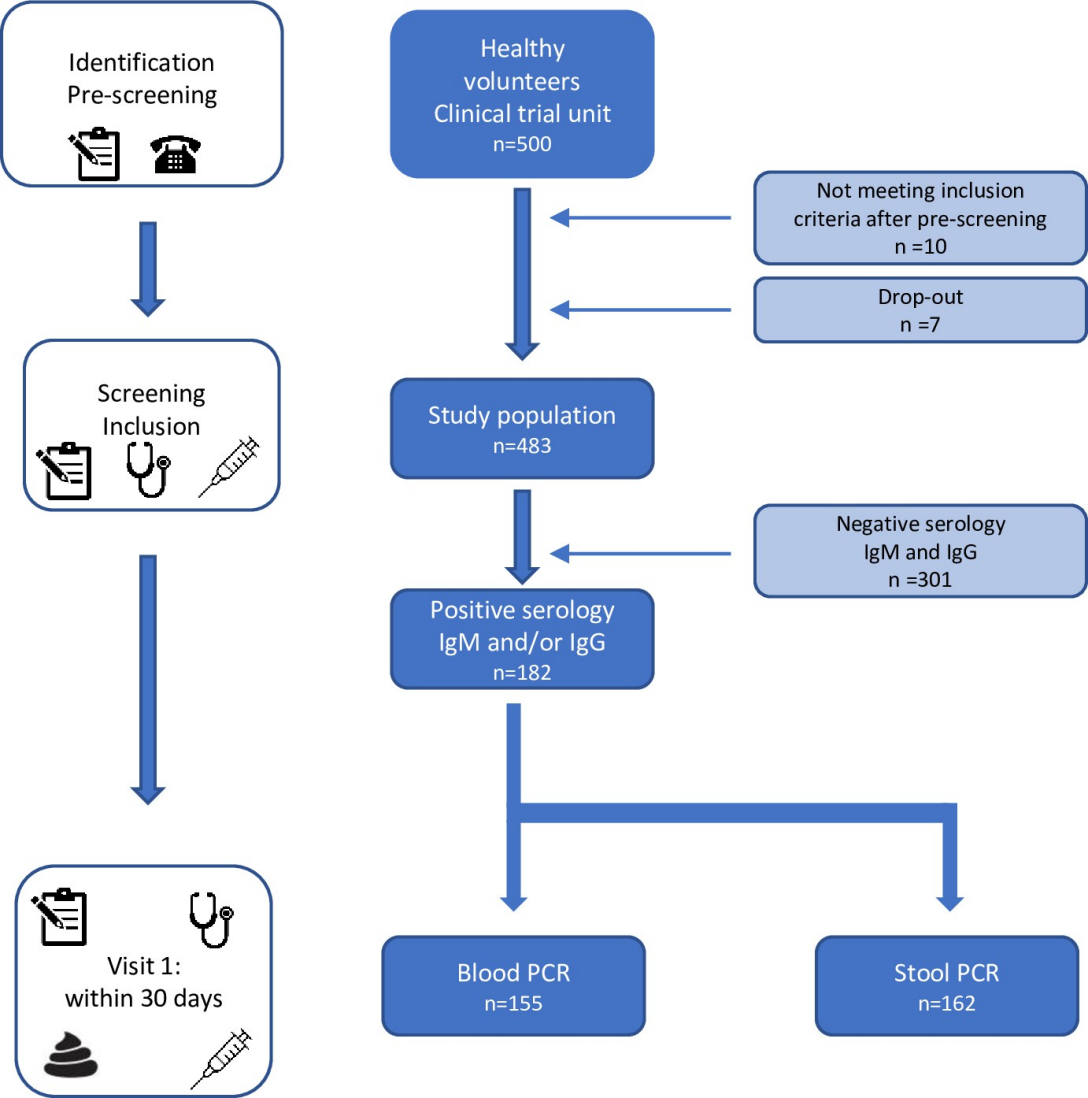
Flowchart of the study. 500 healthy volunteers were enrolled, 483 were included in the study population, 182 had a positive serology.

Five hundred healthy volunteers (250 per center) were enrolled. Of the 500 volunteers, 10 did not meet the inclusion criteria at clinical assessment despite the initial phone screening. Reasons for non-eligibility (one or more criteria) were: abnormal intestinal transit (n = 2), abnormal BMI (n = 2), chronic disease (n = 4), long term medical treatment (n = 3), antibiotic exposition within 3 months (n = 1), living in tropical areas in the 3 months prior enrollment (n = 1), long-term residence in tropical areas (n = 1). Seven participants further dropped out the study after inclusion (Fig 1). Considering the 483 included patients, mean age was 32+10.5 years, there was more females (60.5%) than males, and BMI was 22.9+ 2.9 (Table 1).

**Table 1:** Clinical characteristics of the donors’ population.

**Characteristics**	**Included patients**	**CMV positive**		**CMV negative**
	***n = 483***	***n = 182***		**n = 301**
Age, years mean (SD)	32 (10.5)	33.3 (10.7)		31.1 (10.3)
**Sex** *n* (%)				
• Female	290 (60.5%)	119 (64.7)		173 (57.5)
• Male	193 (39.5%)	65 (35.3)		128 (42.5)
**Body Mass Index**	22.9 (2.9)	23.0 (2.9)		22.8 (2.9)
		**Performed**	**Not performed** *	
**PCR CMV blood** ^**1**^		n = 155	n = 27	
** • negative** ** • positive** ** • uninterpretable**		154 0 1		
**PCR CMV stool**		**Performed** **n = 162**	Not performed** **n = 20**	
** • negative** ** • positive first PCR** ** • positive control second PCR** ** • positive third control PCR**		160 (98.7%) 2 (1.3%) 0 0		

Cytomegalovirus (CMV). Standard deviation (SD); Polymerase chain reaction (PCR)

* No blood samples

** No stool samples available within 30 days

### CMV serology

A total of 483 serological statuses was analyzed: 301(62,3%) were negative for CMV IgG and IgM, and 182 (37,7%) were positive (IgG and/or IgM). Eleven (2,3%) were positive for IgM detection: one presented a primary infection profile (IgG negative), and 10 had detectable IgM, and IgG. Globally, 182 volunteers with CMV IgG or/and IgM positive were eligible for PCR CMV testing in stool and blood specimen. Results are summarized in Table 2.

**Table 2:** CMV serology tests results.

	Included patients	IgM-/IgG-	IgM-/IgG+	IgM+/IgG-	IgM+/IgG+
**Total (n)**	483	301	171	1	10

Immunoglobin M (IgM); Immunoglobulin G (IgG)

### Detection of CMV DNA in whole blood specimens

Blood CMV PCR was performed on 155 volunteers (no blood samples available n = 27). With the exception of one uninterpretable result due to an extraction problem, all results were negative.

### Detection of CMV DNA in stool specimens

Twenty participants with positive CMV serology discontinued the study. The reasons were lost of follow-up between the 2 visits (n = 6), delay > 30 days for stool collection (n = 2), herpes eruption (n = 1), respiratory tract infection (n = 2), pregnancy (n = 2), non-conformity of samples (n = 6), withdrawal of informed consent (n = 1).

CMV PCR was finally performed on 162 volunteers’ stool samples. CMV PCR was negative in 160 volunteers. Six samples were retested due to the detection of inhibitors on the initial test. All were found to be negative, with no inhibitors on the second test. In two cases, the first analysis was positive but below the limit of quantification. The control with PCR CMV versant Siemens and Altostar were negative in the two samples.

### Detection of infectious CMV in cell-culture

All samples were PCR-negative; however, we processed six samples, from IgM-positive volunteers and the two samples with the first PCR positive, in cell culture. No toxicity was observed, and with these methods currently used to isolate viruses from stools, none of the six sample was positive for the detection of the infectious virus through immediate early antigens detection or for classical virus isolation in 25cm^2^flasks.

## Discussion

Cytomegalovirus serology is currently recommended in the screening procedure for fecal microbiota transplantation (FMT) donors [8–10]. Our results show that no CMV DNA was detected by RT-PCR in healthy volunteers’ stools with a positive CMV serology, even in the presence of IgM. CMV DNA PCR in the blood was also negative, and in vitro cell toxicity was also absent. This study is the first to observe that the virus is not detected in the stools or blood of healthy CMV-positive volunteers. This study challenges the necessity of performing this test in the screening process.

CMV transmission is exclusively human-to-human, with humans being the only reservoir. Adult transmission (primo-infection or reinfection) most often occurs through young children’s saliva or urine. Transmission has also been described via sexual route, through milk during breastfeeding, or through transfusion (leuco-reduced blood products have significantly reduced the risk) and solid organ transplant (donor positive- receiver negative) [11–15].

CMV serology status is still recommended in several guidelines, especially in immunocompromised recipients [8, 16]. Only one publication mentioned a possible CMV transmission after FMT [17]. A 37-year-old man known for ulcerative colitis was admitted for abdominal pain with bloody diarrhea. He presented fluctuating abdominal symptoms for the last two months. He had performed four FMTs several weeks before admission, at home without medical supervision. Donors were his wife and a 10-month-old child, whose CMV status was unknown. The final diagnosis was consistent with ulcerative colitis with superimposed cytomegalovirus colitis (compatible histological diagnosis obtained from biopsies of the sigmoid colon and CMV PCR positive in biopsies). No CMV status from the recipient was available on admission or in his past medical history. Thus, it is impossible to know whether the episode was a reactivation or a primary infection. In this case report, CMV transmission from the donor to the patient cannot be established.

A primary CMV infection was described in a randomized clinical trial evaluating the effect of FMT from voluntary donors versus autologous FMT in patients with ulcerative colitis. This observation occurred in the autologous FMT arm (patient’s stool) [18]. Thus, this primary infection was not related to the FMT. To date, no other publication describes a possible CMV transmission via fecal oral route or post-FMT, including in countries or clinical studies that do not recommend performing CMV serology in donors [7].

Even for immunosuppressed individuals who are CMV seronegative, CMV transmission via FMT has never been reported. Data on the safety of FMT with more than ten years of follow-up, including clinical trial monitoring, stool banks, and registries, confirm the low risk of transmission of infectious agents despite the heterogeneous screening methods used [19]. Removing CMV screening will decrease cost related to screening, and will simplify the donor/recipient pathway increasing as direct consequence the number of eligible donors.

However, our study has several limitations. Firstly, due to the lower-than-expected CMV prevalence in the French general population, the number of healthy volunteers with positive CMV serology was lower than anticipated (26% instead of 40–50%). Additionally, we used PCR to detect CMV DNA in stool specimens, which is more sensitive than culture. However, potential PCR inhibitors in feces may be observed. All samples with PCR inhibitors were found to be negative with the second test. Before the study’s initiation, we also compared two methods of pre-analytical stool treatment (centrifugation lysis or BioMérieux) before extracting nucleic acids from dilutions of the WHO CMV standard. For the analysis of healthy volunteers’ stool, we chose the BioMérieux method, which eliminated as many inhibitors as possible while maintaining equivalent sensitivity. False negatives below the PCR detection threshold cannot be excluded.

## Conclusion

Our study aimed to evaluate the prevalence of CMV detection in seropositive healthy donors and did not show any excretion in feces using CMV PCR and culture. This finding reinforces the need to reconsider donor screening, establish a surveillance system through a registry that includes donors, and adopt a uniform method for evaluating the causal relationship of events in FMT-treated patients. In this regard, CMV detection appears to be more of a phantom than a threat.

## Supporting information

S1 File (DOCX)

## References

[1] Kassam Z, Dubois N, Ramakrishna B, Ling K, Qazi T, Smith M, et al. Donor Screening for Fecal Microbiota Transplantation. N Engl J Med. 2019;381: 2070–2072. doi: 10.1056/NEJMc1913670 31665572

[2] Terveer EM, Beurden YH, Goorhuis A, Seegers JFML, Bauer MP, Nood E van, et al. How to: Establish and run a stool bank. Clin Microbiol Infec. 2017;23: 924–930. doi: 10.1016/j.cmi.2017.05.015 28529025

[3] Paramsothy S, Borody TJ, Lin E, Finlayson S, Walsh AJ, Samuel D, et al. Donor Recruitment for Fecal Microbiota Transplantation. Inflamm Bowel Dis. 2015;21: 1600–6. doi: 10.1097/MIB.0000000000000405 26070003

[4] Zhang S, Chen Q, Kelly CR, Kassam Z, Qin H, Li N, et al. Donor screening for fecal microbiota transplantation in China: Evaluation of 8,483 candidates. Gastroenterology. 2021;162: 966–968.e3. doi: 10.1053/j.gastro.2021.11.004 34752816

[5] Tariq R, Weatherly R, Kammer P, Pardi DS, Khanna S. Donor Screening Experience for Fecal Microbiota Transplantation in Patients With Recurrent C. difficile Infection. J Clin Gastroenterol. 2018;52: 146–150. doi: 10.1097/MCG.0000000000000768 27984397

[6] DeFilipp Z, Bloom PP, Soto MT, Mansour MK, Sater MRA, Huntley MH, et al. Drug-Resistant E. coli Bacteremia Transmitted by Fecal Microbiota Transplant. New Engl J Med. 2021;381: 2043–2050. doi: 10.1056/nejmoa1910437 31665575

[7] Lai CY, Sung J, Cheng F, Tang W, Wong SH, Chan PKS, et al. Systematic review with meta‐analysis: review of donor features, procedures and outcomes in 168 clinical studies of faecal microbiota transplantation. Aliment Pharm Therap. 2019;49: 354–363. doi: 10.1111/apt.15116 30628108

[8] Cammarota G, Ianiro G, Tilg H, Rajilic-Stojanovic M, Kump P, Satokari R, et al. European consensus conference on faecal microbiota transplantation in clinical practice. Gut. 2017;66: 569–580. doi: 10.1136/gutjnl-2016-313017 28087657 PMC5529972

[9] Sokol H, Galperine T, Kapel N, Bourlioux P, Seksik P, Barbut F, et al. Faecal microbiota transplantation in recurrent Clostridium difficile infection: Recommendations from the French Group of Faecal microbiota Transplantation. Digest Liver Dis. 2016;48: 242–7. doi: 10.1016/j.dld.2015.08.017 26433619

[10] Woodworth MH, Neish EM, Miller NS, Dhere T, Burd EM, Carpentieri C, et al. Laboratory Testing of Donors and Stool Samples for Fecal Microbiota Transplantation for Recurrent Clostridium difficile Infection. J Clin Microbiol. 2017;55: 1002–1010. doi: 10.1128/JCM.02327-16 28077694 PMC5377826

[11] Cannon MJ, Schmid DS, Hyde TB. Review of cytomegalovirus seroprevalence and demographic characteristics associated with infection. Rev Méd Virol. 2010;20: 202–213. doi: 10.1002/rmv.655 20564615

[12] Fowler KB, Pass RF. Risk Factors for Congenital Cytomegalovirus Infection in the Offspring of Young Women: Exposure to Young Children and Recent Onset of Sexual Activity. Pediatrics. 2006;118: e286–e292. doi: 10.1542/peds.2005-1142 16847076

[13] Stowell JD, Forlin-Passoni D, Din E, Radford K, Brown D, White A, et al. Cytomegalovirus Survival on Common Environmental Surfaces: Opportunities for Viral Transmission. J Infect Dis. 2012;205: 211–214. doi: 10.1093/infdis/jir722 22116837 PMC3276241

[14] Amin MM, Stowell JD, Hendley W, Garcia P, Schmid DS, Cannon MJ, et al. CMV on surfaces in homes with young children: results of PCR and viral culture testing. BMC Infect Dis. 2018;18: 391. doi: 10.1186/s12879-018-3318-z 30103693 PMC6088405

[15] Ziemann M, Thiele T. Transfusion‐transmitted CMV infection–current knowledge and future perspectives. Transfus Med. 2017;27: 238–248. doi: 10.1111/tme.12437 28643867

[16] Kotton CN, Kumar D, Caliendo AM, Huprikar S, Chou S, Danziger-Isakov L, et al. The Third International Consensus Guidelines on the Management of Cytomegalovirus in Solid-organ Transplantation. Transplantation. 2018;102: 900–931. doi: 10.1097/TP.0000000000002191 29596116

[17] Hohmann EL, Ananthakrishnan AN, Deshpande V. Case Records of the Massachusetts General Hospital. Case 25–2014. A 37-year-old man with ulcerative colitis and bloody diarrhea. New Engl J Medicine. 2014;371: 668 675. doi: 10.1056/NEJMcpc1400842 25119613

[18] Rossen NG, Fuentes S, Spek MJ van der, Tijssen JG, Hartman JHA, Duflou A, et al. Findings From a Randomized Controlled Trial of Fecal Transplantation for Patients With Ulcerative Colitis. Gastroenterology. 2015;149: 110–118.e4. doi: 10.1053/j.gastro.2015.03.045 25836986

[19] Marcella C, Cui B, Kelly CR, Ianiro G, Cammarota G, Zhang F. Systematic review: the global incidence of faecal microbiota transplantation‐related adverse events from 2000 to 2020. Aliment Pharm Therap. 2021;53: 33–42. doi: 10.1111/apt.16148 33159374

