# Supplementary material for: Cytomegalovirus in donors for fecal microbiota transplantation, the phantom menace?
Source: PLoS One. 2023 Jun 29;18(6):e0287847. doi: 10.1371/journal.pone.0287847 (PMC10310004; doi:10.1371/journal.pone.0287847)
Supplement: S1 File — (DOCX) [file pone.0287847.s001.docx]

**Supplementary data**

**TRANSFECMV**

**MEDICAL QUESTIONNAIRE FOR PRE-SELECTION**

According to his assessment, this questionnaire serves as a support for the investigating physician to judge whether or not to include the healthy volunteer in the study.

The questions in **bold type** are strict criteria for **not including** the volunteer in the study,

**if the answer given is yes**.

- LAST NAME: FIRST NAME: YEAR OF BIRTH:

- HEIGHT: WEIGHT: Body Mass Index (BMI):

- Do you work as a healthcare provider or in a prison setting? □ yes □ no

- Have you ever participated in a blood donation? □ yes □ no

If yes, when: If refused, what was the reason for refusal:

- Are you or have you been monitored for high blood pressure or high cholesterol,

□ yes □ no

If yes, specify:

**- Are you or have you been followed for any other chronic disease (for example:**

**asthma, diabetes, cancer, hepatitis, autoimmune disease...)? □ yes □ no**

**If yes, specify:**

**- Do you have a history of typhoid fever? □ yes □ no**

- Have you ever had surgery? □ yes □ no

If yes, specify:

- Have you had an organ, tissue (e.g. cornea), or hair transplant? □ yes □ no

**- Have you taken any treatment such as aciclovir, valaciclovir, ganciclovir,**

**valganciclovir, foscarnet, or cidofovir in the past three months? □ yes □ no**

- Do you have any known allergy(s)? □ yes □ no

If yes, specify:

- Have you ever taken growth hormones? □ yes □ no

- Have you ever had a blood exposure accident (contact with human blood by

puncture, wound, splash)? □ yes □ no

If yes, when?

- Is there a case(s) of Creutzfeld-Jacob disease in your family? □ yes □ no

- Is/are there any case(s) of digestive pathology in your family

(e.g. colorectal cancer, bowel polyps, chronic inflammatory

bowel disease, Irritable bowel syndrome)? □ yes □ no

If yes, specify:

**- Do you have chronic bowel disease (ulcerative colitis,**

**Crohn's disease, irritable bowel syndrome)? □ yes □ no**

**If yes, specify:**

- Do you have bowel problems (constipation, diarrhea, abdominal pain, bloating)? □ yes □ no

**If yes, specify:**

**- Have you experienced these problems in the past three months? □ yes □ no**

- Have you recently (within the last three months) taken any treatment that alters transit

(e.g., laxative dose)? □ yes □ no

If yes, specify:

**- Are you taking a long course of treatment (excluding hormonal contraception)?**

**□ yes □ no**

**- Have you taken any antibiotic(s) in the past three months? □ yes □ no**

- Have you taken a probiotic(s) in the past three months? □ yes □ no

- Have you resided in a foreign country? □ yes □ no

If yes, specify the country(es), date and duration:

**- Have you been hospitalized abroad in the past 12 months? □ yes □ no**

- Have you travelled abroad in the past three months? □ yes □ no

If yes, specify:

- Have you used any drug(s) in the past three months? □ yes □ no

If yes, specify:

- Have you had a tattoo, piercing, or acupuncture sessions in the past 3

months? □ yes □ no

If yes, specify the date and location:

- Have you had risky sexual intercourse(s) (new partner or

multiple partners, same-sex relationship) in the past three months? □ yes □ no
